# Breast Hamartoma With Synchronous Contralateral Breast Cancer: A Case Report

**DOI:** 10.7759/cureus.66534

**Published:** 2024-08-09

**Authors:** Zakia El Yousfi, Fatima Zahrae El Mansoury, Asaad El Bakkari, Youssef Omor, Rachida Latib

**Affiliations:** 1 Radiology Department, Ibn Sina University Hospital Center, Rabat, MAR; 2 Radiology Department, Pediatric Teaching Hospital Mohammed V University, Rabat, MAR; 3 Radiology Department, National Institute of Oncology, Rabat, MAR

**Keywords:** breast within breast sign, pten hamartoma tumor syndrome, synchronous breast cancer, imaging, breast hamartoma

## Abstract

Breast hamartoma is a rare benign growth often overlooked and consequently not well-documented, mainly due to insufficient recognition of its distinct clinical and histological features. Increasing awareness about this relatively obscure benign condition is crucial because it can mimic both benign and malignant breast tumors clinically. Its association with breast cancer is infrequently documented in medical literature. Additionally, it may be linked to PTEN hamartoma tumor syndrome, which involves a mutation of the *PTEN* tumor suppressor gene. This article presents a case study of a young woman diagnosed with left breast carcinoma, where imaging revealed a sizable mass on the opposite breast consistent with a breast hamartoma.

## Introduction

Breast hamartoma presents as a well-defined, encapsulated nodule comprising disorganized breast tissue components. Various variants exist, characterized by combinations of glandular and fibroadipose elements such as adenolipoma, fibroadenolipoma, and myoid hamartoma [1]. The exact pathogenesis of breast hamartoma remains unclear. Pathologically, hamartomas lack distinct features. Clinically, they are often discovered incidentally during mammographic screening and are considered underdiagnosed. However, with the increased use of breast imaging modalities such as ultrasound, mammography, core needle biopsy, and fine needle aspiration cytology, the identification of breast hamartomas is on the rise. It is critical to highlight that these lesions can be easily overlooked without thorough clinical or radiological examination. Their association with breast cancer is seldom documented in the literature and may be linked to PTEN hamartoma tumor syndrome, involving mutations in the *PTEN *tumor suppressor gene [2].

## Case presentation

**Figure 1:**
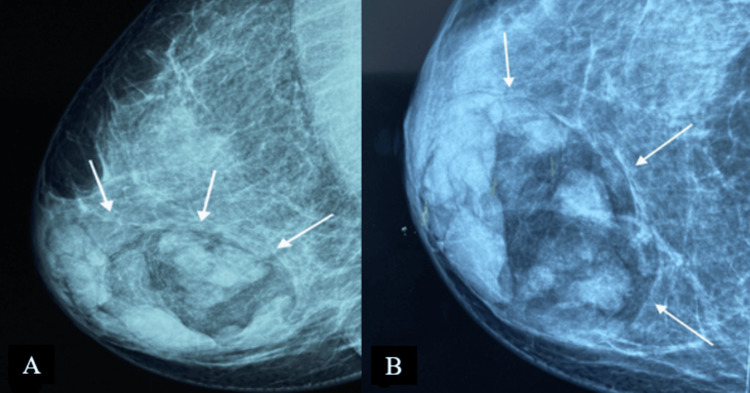
Digital mammography: medio-lateral oblique (A) and cranio-caudal (B) projections of the right breast A large, well-defined mass containing radiolucent (fat) and radiopaque (soft tissue) densities can be seen. It is surrounded by a thin radiopaque capsule, presenting a “breast within a breast” appearance. This mass is classified as BI-RADS 2.

A 37-year-old woman, with no personal or family history of breast disease, was admitted with a diagnosis of invasive ductal carcinoma of the left breast. She also presented with a large palpable mass in the right breast. Mammography revealed a sizable, well-circumscribed mass with a capsule, showing heterogeneous central density containing both radiopaque and radiolucent areas, classified as BI-RADS (Breast Imaging-Reporting and Data System) 2 (Figure 1).

**Figure 2:**
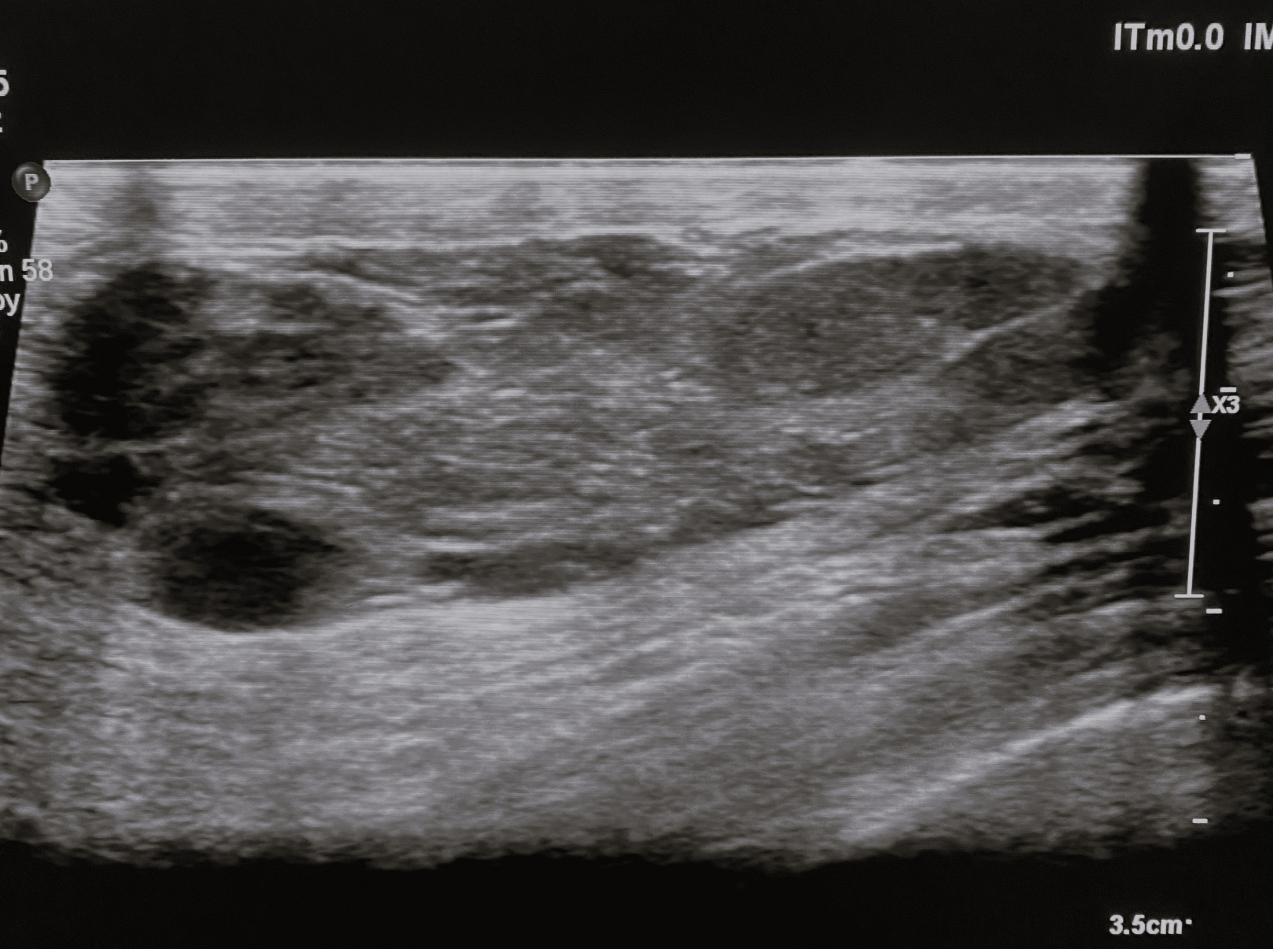
Breast ultrasound Breast ultrasound shows a tissue formation with the long axis parallel to the cutaneous plane, well-circumscribed, heterogeneous, with hypoechoic with hyperechoic trabeculae and posterior acoustic enhancement, designated as BI-RADS 2.

Ultrasound showed a well-defined lesion with heterogeneous echogenicity, displaying hypoechoic and hyperechoic bands (Figure 2).

**Figure 3:**
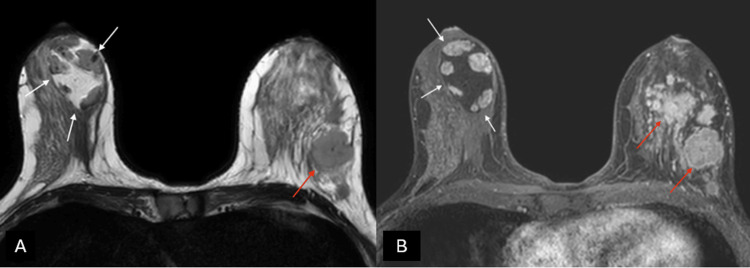
(A) Axial T2-weighted images and (B) T1-weighted fat-suppressed enhanced magnetic resonance imaging (MRI) A capsulated, large-sized hamartoma can be seen located in the outer quadrants of the right breast, exhibiting a signal identical to normal mammary gland tissue, creating a “breast within a breast” appearance, classified as BI-RADS 2 (white arrows). There are multiple masses in the contralateral breast with suspicious morphology, associated with a known invasive ductal carcinoma, classified as BI-RADS 6 (red arrows).

Given the neoplastic context, breast MRI was performed to assess for multifocality, which confirmed a BI-RADS 6 classification for the left breast, and identified a large mass in the right breast demonstrating the classic "breast within a breast" sign, containing adipose tissue and variably enhanced matrix corresponding to a right breast hamartoma, classified as BI-RADS 2 (Figure 3).

## Discussion

The term "hamartoma," originating from Greek meaning "bodily defect," refers to an abnormal mixture of native tissues at a specific location. Hamartomas are benign, non-cancerous growths that can recur and are found in various parts of the body, including the lungs, kidneys, and breasts. Breast hamartomas are composed of a heterogeneous blend of glandular epithelial, fibrous, and adipose tissues, constituting approximately 4.8% of all benign breast lesions [1].

The precise cause of breast hamartoma development remains unclear, although it is believed to be a developmental anomaly rather than a true neoplastic process. Estrogen receptors and progesterone receptors expressed by the epithelial and stromal components of breast hamartomas are thought to play a role [3].

These growths typically manifest in premenopausal women, most commonly in their forties but spanning a broad age range from adolescents to women in their eighties. Breast hamartomas are often underdiagnosed, but the increased use of diagnostic tools for breast masses has led to more frequent identification [4].

Clinically, breast hamartomas present as painless, movable, firm-to-soft masses typically located in the outer quadrants of the breast [5]. This clinical presentation may lead physicians to consider differential diagnoses such as fibroadenomas and other tumors. Nearly 60% of breast hamartomas are not palpable and are detected through radiological imaging. Diagnostic tools such as ultrasound, mammography, magnetic resonance imaging (MRI), needle biopsy, and fine needle aspiration cytology are valuable in confirming the diagnosis of breast hamartomas.

On mammography, breast hamartomas present with varying appearances influenced by the relative proportions of their components. They typically exhibit a well-defined outline, containing both fatty tissue and dense nodules of soft tissue, enclosed within a thin radiopaque capsule formed by displaced breast parenchyma. Occasionally, hamartomas may appear uniformly dense if rich in fibrous tissue, posing a challenge in differentiation from fibroadenomas, which also display uniform density and often a thin radiolucent capsule compressed by fat [6].

Ultrasound examination reveals well-defined lesions with smooth borders and internal echogenicity that can be hyperechoic or heterogeneous. Breast hamartomas do not typically show posterior acoustic shadowing, a characteristic ultrasound sign used to distinguish benign from malignant lesions. Some lesions may exhibit a halo separating them from surrounding tissue, primarily echogenic due to condensed breast tissue, but may also appear anechoic, likely due to compressed fat between the hamartoma and adjacent breast tissue [7].

On conventional T1- and T2-weighted MRI images, breast hamartomas demonstrate heterogeneous intensities consistent with findings from other imaging modalities, often accompanied by a thin capsule [8]. While needle biopsy and fine needle aspiration cytology are essential for diagnosing most breast lesions, they are less effective for diagnosing hamartomas and differentiating them from mimicking entities such as fibroadenomas. Histological examination, however, can distinguish breast hamartomas by revealing ductal and/or stromal proliferation resulting in intercanalicular or pericanalicular growth patterns. Hamartomas primarily composed of fat may be mistakenly diagnosed as lipomas, fatty necrosis, or cysts [1].

Macroscopically, breast hamartomas typically present as round-to-oval lesions that can reach sizes up to 20 cm. On cut surfaces, they often resemble normal breast parenchyma or, depending on consistency, may resemble fibroadenomas. Histologically, hamartomas are mostly encapsulated, either by a true capsule or a peripheral pseudocapsule. The lesion exhibits a lobulated structure and contains variable proportions of breast ducts, lobules, fibrous tissue, and adipose tissue [3].

Although rare, breast hamartomas have been occasionally associated with malignancies. A few cases have been documented in the literature of breast cancers arising within hamartomas [9].

To our knowledge, this is the second reported case of breast parenchymal hamartoma with synchronous contralateral breast cancer. The first case involved a myoid breast hamartoma with synchronous contralateral breast cancer [10]. This association may be related to PTEN hamartoma tumor syndrome, characterized by mutations in the PTEN tumor suppressor gene. This syndrome increases the lifetime risk of breast cancer and the development of hamartomas in various tissues, underscoring the need for genetic counseling [2].

Currently, surgical excision is considered curative for breast hamartomas. They generally have an excellent prognosis whether or not they are surgically removed. While some suggest observation without excision if confidently diagnosed by mammography [11], surgical removal is typically recommended due to reports of malignancies associated with these lesions. Given their well-circumscribed nature and often clear borders, hamartomas are usually easily enucleated. After surgery, patients are advised to undergo mammography and ultrasound every six months for one to two years to monitor for stability and potential recurrence.

## Conclusions

Breast hamartoma is a rare benign lesion composed of a mixture of normal breast tissues, such as glandular, adipose (fatty), and fibrous tissues. It tends to be overlooked and therefore not frequently reported. However, with the rise in the use of breast diagnostic techniques such as ultrasound and mammography, diagnoses of breast hamartomas are increasing. Malignancies linked with breast hamartomas are infrequent, and surgical removal is typically curative. Clinicians, particularly radiologists and surgeons, should recognize this condition as a potential differential diagnosis for breast masses, especially those with well-defined boundaries.
